# Medium Optimization for the Production of Fibrinolytic Enzyme by *Paenibacillus* sp. IND8 Using Response Surface Methodology

**DOI:** 10.1155/2014/276942

**Published:** 2014-01-09

**Authors:** Ponnuswamy Vijayaraghavan, Samuel Gnana Prakash Vincent

**Affiliations:** International Centre for Nanobiotechnology, Centre for Marine Science and Technology, Manonmaniam Sundaranar University, Rajakkamangalam, Kanyakumari District, Tamil Nadu 629 502, India

## Abstract

Production of fibrinolytic enzyme by a newly isolated *Paenibacillus* sp. IND8 was optimized using wheat bran in solid state fermentation. A 2^5^ full factorial design (first-order model) was applied to elucidate the key factors as moisture, pH, sucrose, yeast extract, and sodium dihydrogen phosphate. Statistical analysis of the results has shown that moisture, sucrose, and sodium dihydrogen phosphate have the most significant effects on fibrinolytic enzymes production (*P* < 0.05). Central composite design (CCD) was used to determine the optimal concentrations of these three components and the experimental results were fitted with a second-order polynomial model at 95% level (*P* < 0.05). Overall, 4.5-fold increase in fibrinolytic enzyme production was achieved in the optimized medium as compared with the unoptimized medium.

## 1. Introduction

Fibrin is the main protein component of the blood clot, and it is normally formed from fibrinogen by the action of thrombin (EC. 3. 4. 21. 5) after trauma or injury. Accumulation of fibrin in blood vessels usually increases thrombosis, leading to myocardial infarction and other cardiovascular diseases (CVDs). A variety of fibrinolytic enzymes such as tissue plasminogen activators (t-PA), urokinase (u-PA), and streptokinase were extensively studied and used as thrombolytic agents [1]. Although t-PA and u-PA are still widely used in thrombolytic therapy today, their expensive prices and undesirable side effects prompt researchers to search for cheaper and safer thrombolytic agents. In recent years, fibrinolytic enzymes from various sources, including microorganisms, worms, and animals, have been the subject of active researches because of their potential as novel agents in preventing or treating CVDs by dissolving fibrin blood clot [2, 3]. The genus Bacillus from traditional fermented food is an important one among the microorganisms that have been found to produce the fibrinolytic enzymes [4]. Fibrinolytic enzymes from these food-grade microorganisms can be promising alternatives for t-PA or streptokinase in thrombolytic therapy. Nattokinase, a potent fibrinolytic enzyme, has been reported to have a potent thrombolytic activity [5]. Based on its food origin and relatively strong fibrinolytic activity, nattokinase has advantages over other commercially used medicine, in preventative and prolonged effects, convenient oral administration, and stability in the gastrointestinal tract [6].

Many fibrinolytic enzymes have been isolated from various foods such as Korean *chungkookjang *[7], Chinese *douche* [8], soybean grits [9], and Indonesian* tempeh* [10]. Endophytic bacteria such as *Paenibacillus *produce biotechnologically important enzymes. The genus *Paenibacillus *was created by Ash et al. [11] to accommodate the “group 3” of the genus *Bacillus*. It comprises over 30 species of facultative anaerobes and endospore-forming, neutrophilic, periflagellated, heterotrophic, and low G + C Gram-positive bacilli. The name reflects this fact; in Latin *paene *means *almost*, and therefore the *Paenibacillus *is almost a *Bacillus*. Very few studies were carried out in the optimization and characterization of fibrinolytic enzymes from *Paenibacillus* sp. Recently, Lu et al. [12] purified and characterized fibrinolytic enzymes from *Paenibacillus polymyxa* EJS-3.

Moreover, very few studies were reported on statistical optimization of fibrinolytic enzymes production in solid-state fermentation (SSF). Tao et al. [13] optimized process parameters for the production of fibrinolytic enzymes by *Fusarium oxysporum. *Compared to submerged fermentation, SSF yields more enzyme and it could reduce the production cost of the enzyme. From an industrial point of view, around 30–40% of the production cost of enzymes is estimated to account the cost of the growth medium [14]. Therefore, optimization of the fermentation process parameters in SSF through a statistical approach is important for a significant improvement in yield as well as a decrease in the production cost of the enzyme. The selection of medium components is another critical factor for the production of fibrinolytic enzymes because each microbe requires unique nutrient components and environmental conditions for its growth and the production of fibrinolytic enzymes [9, 15]. Since wheat bran was recognized as the standard substrate for SSF, it was selected for the production of fibrinolytic enzymes.

The traditional one-at-a-time optimization strategy is simple, but it fails to locate the region of optimal response because the comprehensive effect of factors is not taken into consideration for the production of fibrinolytic enzymes [16]. The statistical experimental design provides a universal language with which experts from different areas such as academia, engineering, business, and industry can communicate for setting, performing, and analyzing experiments for research. Statistically designed experiments are more effective than other classical one-at-a-time optimization strategy because it can study many variables simultaneously with a low number of observations, saving time, and costs [17, 18]. The statistical method such as factorial design, central composite design and response surface methodology (RSM) were frequently used to optimize the process parameters for the production of antimicrobial metabolites [19], bio-surfactants [20], and fibrinolytic enzymes [16, 21]. The main objective of this study was to optimize the process parameters by statistical approach for enhancing fibrinolytic enzymes production by *Paenibacillus* sp. using two-level full factorial's design followed by RSM.

## 2. Materials and Methods

### 2.1. Isolation of Fibrinolytic Enzymes-Producing Strain

The fibrinolytic enzymes-producing strain-IND8 was isolated from the cooked Indian rice. Samples collected were plated onto skim-milk agar plates containing (g/L) peptone 5, yeast extract 5, NaCl 1.5, agar 15, and skim milk 10. These plates were incubated for 24–48 h at 37°C and a clear zone on skimmed milk hydrolysis gave an indication of protease-producing strains. These protease producing strains were subjected to fibrinolytic enzymes screening. Fibrinolytic enzymes production was carried out in the culture medium composed of (g/L) peptone 5, yeast extract 5, NaCl 1.5, and Casein 10. Medium was autoclaved at 121°C for 20 min and a loopful culture of the selected organism was inoculated. Submerged fermentation was performed on a rotary shaker (150 rpm) for 48 h at 37°C, in 250 mL Erlenmeyer flasks. The cultures were centrifuged and the supernatants were used for determination of fibrinolytic activity using a fibrin plate. The fibrin plate was composed of 1% (w/v) agarose, 0.5% (w/v) fibrinogen, 1% (v/v), and thrombin (100 NIH units/mL) (pH 7.4) [22]. The fibrin plate was allowed to stand for 1 h at room temperature to form a fibrin clot layer. Ten microliters of crude enzyme was dropped into holes and incubated for 5 h at 37°C, fibrinolytic enzymes exhibited a clear zone of degradation of fibrin around the well indicating its activity. The single-strain IND8 showing the largest halo zone on the fibrin plate was selected and further identified.

### 2.2. S rDNA Sequencing

The genomic DNA was extracted from the cells of an 18 h culture using QIAGEN genomic DNA purification kit according to the manufacturer's instructions. The 16s rDNA gene was amplified by PCR (Peltier Thermal Cycler Machine, USA) using the upstream (P1: ^5′^AGAGTTTGATCMTGGCTAG^3′^) and the downstream primers (P2: ^5′^ACGGGCGG  TGTGTRC^3′^) and DNA polymerase (Sigma, USA). The amplified product was sequenced and sequence comparison with the databases was performed using BLAST through the NCBI server [23]. The 830 bp 16S rDNA sequences of *Paenibacillus* IND8 strain were submitted to GenBank database under an accession number KF250416.

### 2.3. Assay of Fibrinolytic Enzymes Activity

The culture supernatant (0.1 mL) suitably diluted was mixed with 2.5 mL of 0.1 M Tris-HCl buffer (pH 7.8) containing 0.01 M calcium chloride. To this, 2.5 mL of fibrin (1.2%, w/v) was added and incubated for 15 min at 37°C. The reaction was stopped by adding 5.0 mL of 0.11 M trichloroaceticacid containing 0.22 M sodium acetate and 0.33 M acetic acid. The absorbance was measured at 275 nm against sample blank. A standard curve was performed using L-tyrosine. One unit of fibrinolytic activity was defined as the amount of enzyme which liberates 1 *μ*g of tyrosine per minute under the experimental conditions used.

### 2.4. Fibrin Zymography and *In Vitro* Analysis of Blood Clot

Fibrin zymography was carried out in 12% SDS-polyacrylamide gel containing fibrinogen (0.12%, w/v) and 100 *μ*L thrombin (10 NIH units/mL). After electrophoresis at 4°C, the gel was incubated in 0.05 M sodium phosphate buffer (pH 7.4) containing 2.5% triton X-100 for 30 min at room temperature. Further, the gel was washed with distilled water for 30 min and incubated in sodium phosphate buffer (pH 7.4, 0.05 M) for 5 h at 37°C. It was stained with coomassie brilliant blue R-250 for 1 h, after which it was destained and bands with fibrinolytic activities were visualized as the nonstained region of the gel. Clot lytic effects of fibrinolytic activities were studied with natural clot *in vitro*. The goat blood clot was cut into the same size, and crude fibrinolytic enzymes were added. The mixture was incubated at room temperature for 24 h and analyzed for its activities on fibrin blood clot [24].

### 2.5. Primary Screening of Process Parameters Based on One-at-a-Time Strategy

In the present study, SSF was carried out using wheat bran as a substrate. SSF was carried out separately in a 100 mL Erlenmeyer flask containing 2.0 g (w/w) of the substrate moistened with 2.0 mL buffer (pH 8.0, 0.1 M). The contents were sterilized and inoculated with 0.2 mL of 18 h grown (0.796 OD at 600 nm) culture broth under sterile conditions. The process parameters such as the fermentation period (24–96 h), pH (6.0–10.0), moisture content (60%–140%), inoculum size (3%–15%), carbon sources (1%, w/w) (maltose, sucrose, starch, glucose, xylose, and trehalose), nitrogen sources (1%, w/w) (casein, yeast extract, peptone, beef extract, gelatin, and urea), and inorganic salts (ammonium chloride, sodium dihydrogen phosphate (NaH_2_PO_4_), calcium chloride, sodium nitrate, disodium hydrogen phosphate (Na_2_HPO_4_), ammonium sulphate, and ferrous sulphate) were evaluated. Twenty milliliters of double distilled water was added with the fermented medium and enzyme extracted as described earlier [25]. All experiments were carried out in triplicate, and average values are reported.

### 2.6. Evaluation of Significant Factors Affecting Fibrinolytic Enzymes Production by 2^5^ Factorial Designs

Two-level full factorial designs were carried out for screening the most significant factors affecting the fibrinolytic enzymes production by *Paenibacillus* IND8. Five factors, namely, sucrose (carbon source), yeast extract (nitrogen source), NaH_2_PO_4_ (inorganic salt), pH, and moisture content of the medium were selected for the analysis of significant factors. Based on two-level full factorial design each factor was examined at two-levels (− and +). The other factors such as fermentation period and inoculum were kept at middle level. Two-level full factorial designs were based on the following first-order polynomial model. Consider

(1) $$\begin{array}{c} Y=\alpha_{0}+\sum_{i}^{}\alpha_{i}x_{i}+\sum_{ij}^{}\alpha_{ij}x_{i}x_{j}+\sum_{ijk}^{}\alpha_{ijk}x_{i}x_{j}x_{k}, \end{array}$$

where *Y* is the response (fibrinolytic activity). *α*_*ij*_ and *α*_*ijk*_ were the *ij*th and *ij* *k*th interaction coefficients; *α*_*i*_ was the *i*th linear coefficient and *α*_0_ was an intercept.

Fibrinolytic activity assay was carried out in triplicates and the average of these experimental values was taken as response *Y*. ANOVA was used to estimate the statistical parameters and the values of “Prob > *F*” less than 0.05 indicated that the model terms are significant. Statistical software, Design-Expert 8.0.7.0 (StatEase Inc, Minneapolis, USA), was used to design the experiment. Experimental design and results of the 2^5^ factorial designs were described in Table 1. The significant factors (*P* < 0.05) obtained from two-level full factorial designs were further optimized by RSM.

### 2.7. Central Composite Design and Response Surface Methodology

Central composite design was employed in the present investigation to estimate the main effects. The factors used were sucrose, NaH_2_PO_4_, and moisture for enhanced fibrinolytic enzymes production. Each factor in the design was studied at five levels (−*α*, −1,0, +1, and +*α*) in a set of 20 experiments that included 8 factorial, 6 axial, and 6 center points. All experiments were conducted in triplicates and the mean values of fibrinolytic activities (units/mL) were taken as the response (*Y*). The second-order polynomial equation was employed to fit the experimental data. For a three-factor system the second-order polynomial equation is as follows:

(2) $$\begin{array}{c} Y=\beta_{0}+\sum_{I=1}^{3}\beta_{i}X_{i}+\sum_{i=1}^{3}\beta_{ii}X_{i}^{2}+\sum_{ij=1}^{3}\beta_{ij}X_{ij}, \end{array}$$

where *Y* was the response, *β*_0_ was the offset term, and *β*_*i*_, *β*_*ii*_, and *β*_*ij*_ were the coefficients of linear terms, square terms, and coefficients of interactive terms, respectively. *X*_*i*_'s were A, B and C, *X*_*ij*_'s were AB, AC, and BC.

Analysis of variance (ANOVA) was used to estimate the model. The values of “Prob > *F*” less than 0.05 indicated that the model terms were significant. The fitted polynomial equation was expressed as three-dimensional surface plots to visualize the relationship between the responses and the levels of each factors used in the design. The statistical software (Design-Expert 8.0.7.0, StatEase Inc, Minneapolis, USA) was used to plot the 3D graphs.

### 2.8. Statistical Model Validation

With the help of the special features of RSM and 3D graph and perturbation plot the optimum value of the combination of the three factors (sucrose, NaH_2_PO_4_, and moisture) were validated. Experiments were carried out in triplicates in Erlenmeyer flask under theoretically predicted conditions to validate the model.

## 3. Results and Discussion

### 3.1. Bacterial Strain

**Figure 1:**
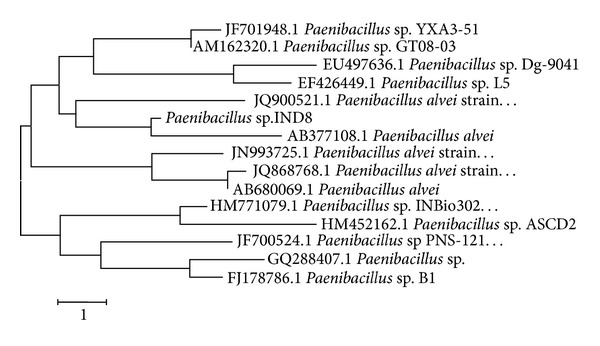
Phylogenetic relationships of stain IND8 and other closely related *Paenibacillus *based on 16S rDNA sequence. Bar = 1 substitution per site.

Among the bacterial isolates strain IND8 was selected for this study in light of exhibiting strong fibrinolytic activities. The isolated strain was Gram-positive rods, citrate-, oxidase-, and nitrate-positive. It was negative to urea-, indole- and gelatin-hydrolysis. It hydrolyzed casein and fermented carbohydrates. The strain-IND8 was identified as *Paenibacillus* sp. based on its 16s rDNA sequence and designated as *Paenibacillus* sp. IND8. The phylogenetic tree constructed from the sequenced data by the neighbor-joining method showed the detailed evolutionary relationship between the strain IND8 and other closely related *Paenibacillus *sp. (Figure 1).

### 3.2. Fibrin Zymography and *In Vitro* Analysis of Fibrinolytic Activities

Fibrin zymography revealed at least three major and two minor fibrinolytic proteases were determined from the crude extract (figure not shown). Fibrin clot degradation was observed within 24 h of incubation at room temperature (30°C) in the tube containing fibrinolytic enzymes. In the saline solution suspend tubes, the blood clot remained (figure not shown). These fibrinolytic enzymes may have wide application in pharmaceutical industry. Fibrinolytic enzymes from this kind of food grade organisms could effectively prevent and treat cardiovascular diseases [26].

### 3.3. Initial Screening of Physical and Nutrient Factors for Statistical Optimization Process

**Figure 2:**
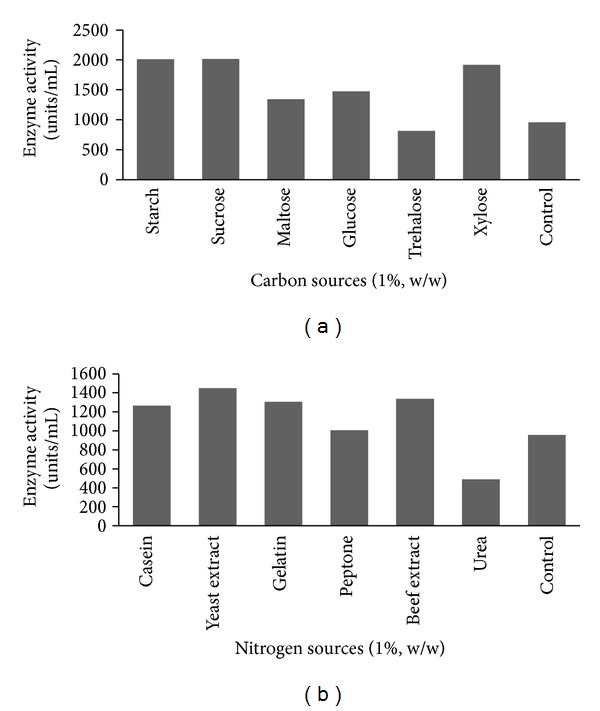
(a) Effect of different carbon sources on production of fibrinolytic enzymes. (b) Effect of different nitrogen sources on production of fibrinolytic enzymes.

The physical and nutrient factors were optimized by a traditional one-factor-at-a-time approach. In order to achieve the maximum yield of fibrinolytic enzymes, the following optimum process parameters are needed: fermentation period (72 h), pH (8.0), moisture (80–100%), and inoculum (6–9%). Among the carbon sources supplemented, sucrose supported more enzymes production (2017 units/mL) than other sources (Figure 2(a)). Among the nitrogen sources, yeast extract showed more enzymes production (1451 units/mL) (Figure 2(b)). Fibrinolytic enzymes production was high in the presence of sodium dihydrogen phosphate as the sole source of an in-organic salt (1630 units/mL). One-factor-at-a-time experiments revealed that sucrose, yeast extract, and sodium dihydrogen phosphate significantly increased fibrinolytic enzymes production. Hence, these nutrient factors were selected for statistical optimization. From the SSF point of view, moisture is one of the critical factors for enzyme production; thus it was selected for statistical optimization. The protease production by microbial strains strongly depends on the extracellular pH [27]. Hence, the pH of the medium was also considered for statistical medium optimization approach.

Conventional experimental approach used for media optimization employing “change-one-factor-at-a-time” is extremely time consuming and expensive and laborious for screening a large number of variables. Optimizing all the significant parameters by statistical experimental designs can eliminate these limitations of a single factor optimization process collectively. Several statistical designs are available such as full factorial, fractional factorial or Plackett-Burman designs, Taguchi's robust designs, and response surface methodology [28]. In this study, the important factors such as sucrose, yeast extract, NaH_2_PO_4_, pH, and moisture were optimized by two-level full factorial designs and response surface methodology.

### 3.4. Two-Level Factorial Designs on Elucidation of Medium Components

The fibrinolytic enzymes production varied from 723 to 3768 units/mL with different combinations of the components supplemented with wheat bran. Table 1 represented the two-level full factorial designs for five selected variables (sucrose, yeast extract, NaH_2_PO_4_, pH, and moisture) and the corresponding response for fibrinolytic enzymes production. The nutrient factors such as, sucrose, yeast extract, and NaH_2_PO_4_ were positively correlated and this indicated that the further increase in the concentrations of these nutrients could increase the production of fibrinolytic enzymes. Among the all nutrient and physical factors, moisture content of the medium was significantly influenced by fibrinolytic enzymes production.

Analysis of variance (ANOVA) was used to evaluate the observed results. The model *F* value of 125.57 implied the model was significant. There is only a 0.01% chance that a “model *F* value” this large could occur due to noise. Values of “Prob > *F*” less than 0.05 indicate that model terms were significant. In this model A, C, D, E, AB, AC, AD, AE, BC, BD, BE, CD, CE, DE, ABC, ABD, ACE, ADE, BCD, BCE, BDE, CDE, ABCD, ABCE, ABDE, ACDE, and ABCDE were highly significant. The correlation coefficient of this model was 0.998. The “predicted *R*-squared” of 0.924 was in reasonable agreement with the “adjusted *R*-squared” of 0.99. “Adequate precision” measures the signal to noise ratio. A ratio greater than 4 is desirable. In this model the ratio of 45.11 indicates an adequate signal. For pH, the coefficient estimate was negative (−95.22) and it indicated that the reduction of pH could positively influence the enzymes production. Yeast extract did not influence the fibrinolytic enzymes production significantly (*P* > 0.05). Neglecting the insignificant variables, the model equation for fibrinolytic enzymes production can be written as

Enzyme activity = +2128.28 + 151.22A + 80.34C − 95.22D + 387.47E + 88.28AB − 158.22AC + 78.22AD + 51.66AE + 214.03BC − 43.66BD + 62.53BE − 293.66CD + 63.16CE − 52.78DE + 46.59ABC + 88.41ABD + 100.34ACE − 178.09ADE − 159.84BCD − 115.28BCE − 55.09BDE − 69.34CDE − 48.28ABCD − 158.22ABCE + 204.97ABDE − 116.91ACDE − 52.84ABCDE.

Enzyme production was found to be high (3768 units/mL) in the wheat bran containing 0.75% sucrose, 0.5% yeast extract, and 0.1% NaH_2_PO_4_ with 100% moisture content at pH 7.0 (run 32). Wheat bran was considered as a standard substrate for the production of proteolytic enzymes and Chang et al. [29] used wheat bran medium for the production of fibrinolytic enzymes by *Bacillus subtilis* IMR-NK1. The two-level full factorial designs showed that the medium containing 100% moisture showed increased fibrinolytic activities. The moisture content of the fermentation medium is one of the main factors in SSF and often determines the success of a process [30]. Based on the experimental result, yeast extract is the best choice of nitrogen source for enzyme bioprocess. These observations were in accordance with the observations made with *Bacillus* sp. [31].

Fibrinolytic activities were found to be high in the presence sucrose with the wheat bran medium. These results were in accordance with reported protease production in the presence of different sugars [27]. Based on the calculated *t* values (Table 2), sucrose, NaH_2_PO_4_, and moisture were selected for further optimization by RSM.

### 3.5. Optimization by Central Composite Designs (CCD) and Statistical Analysis

CCD was used for optimization of three variables: sucrose (A), NaH_2_PO_4_ (B), and moisture (C), each were studied at five coded levels, that is, (−*α*, −1, 0, +1, +*α*) as shown by Table 3 and the models were explained with twenty runs (Table 4).

In the recent years major research and development on the use of statistical methods involving various statistical software packages for the optimization studies with the aim of obtaining high yields of amylases, proteases, biosurfactants, neomycin, and so forth, [32]. Limitations and drawbacks of the single factor optimization can be eliminated by employing RSM which was used to explain the combined effects of all the factors in a fermentation process [33]. The commonly used response surface design was CCD [34] involving five levels, for each factor needed for quadratic terms to be estimable in the second-order model.

In this study the responses of the CCD were well fitted with a second-order polynomial equation.

Fibrinolytic activity (*Y*) = +3728.21 + 204.66A − 509.50B + 927.80C + 33.75AB − 335.00AC + 278.50BC − 344.06A^2^ − 377.65B^2^ − 200.

Here, A-sucrose; B-NaH_2_PO_4_; C-moisture.

The model *F* value of 10.74 implied that the model was significant. There is only a 0.05% chance that a “model *F* value” this large could occur due to noise. Values of “Prob > *F*” less than 0.05 indicated that the model terms were significant. In this model, the linear terms B and C (*P* < 0.05) and quadratic terms A^2^ and B^2^  (*P* < 0.05) were statistically significant (Table 5). The linear factor A, interaction terms (AB, AC, BC), and quadratic term (C^2^) were insignificant (*P* > 0.05). The “lack of fit *F* value” of 2.85 implies the lack of fit is not significant relative to the pure error. There is a 13.75% chance that a “lack of fit *F* value” this large could occur due to noise. Nonsignificant lack of fit is good. “Adequate precision” measures the signal (response) to noise (deviation) ratio. A ratio greater than 4 is desirable. In this model the ratio 12.729 indicates an adequate signal and therefore the model is significant for the enzyme bioprocess.

**Figure 3:**
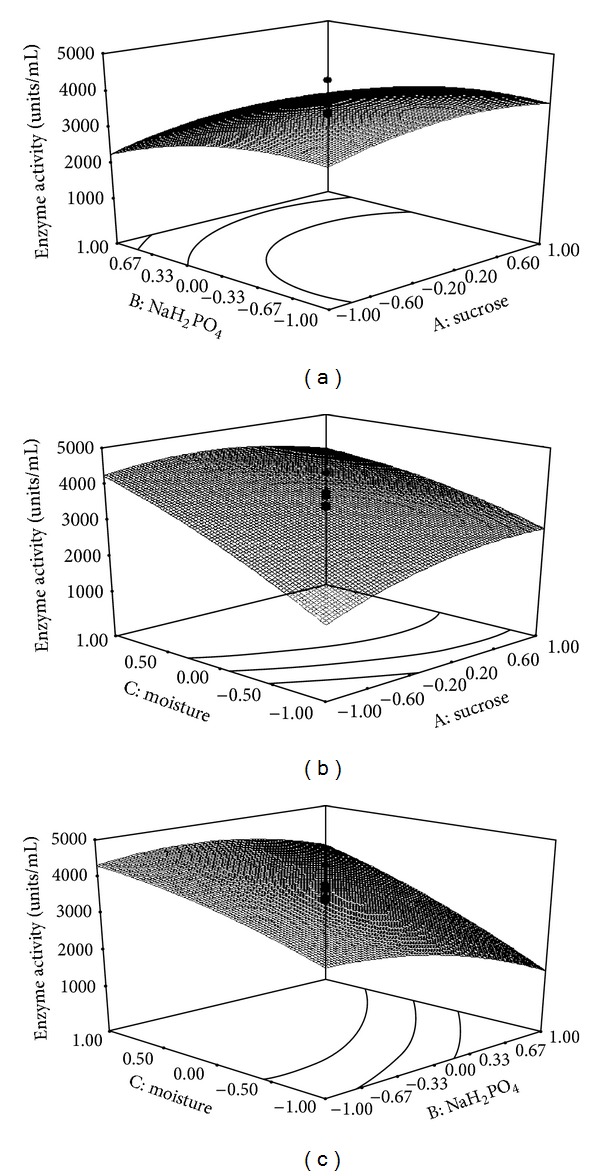
(a)–(c) Response surface plots showing the effect of interaction of sucrose and NaH_2_PO_4_ (a), sucrose and moisture (b), and moisture and NaH_2_PO_4_ (c).

**Figure 4:**
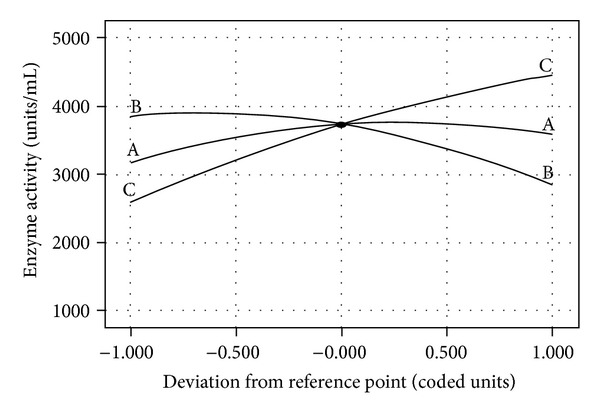
Perturbation graph summarizing the effect of sucrose (A), NaH_2_PO_4_ (B), and moisture (C) on fibrinolytic enzymes production.

The goodness-of-fit of the model was checked by comparing the coefficient of determination (*R* ^2^) with adjusted coefficient of determination (*R* ^2^), which are measures of the amount of the reduction in the variability of response obtained by using the repressor variables in the model [35]. In this model, the coefficient of determination (*R* ^2^) is 0.9063 and it explains 90.63% variability in the model and the adjusted *R* ^2^ was 0.82. The *R* ^2^ value closer to 1.0 shows a stronger model with better predictability [36]. Figures 3(a)–3(c) depicted the 3D plot of the response from the interaction among variables and determined the optimum concentrations of each factor for maximum fibrinolytic enzymes production by *Paenibacillus *sp. Fibrinolytic enzymes production varied significantly upon changing the initial concentrations of NaH_2_PO_4_ and moisture. The three-dimensional plots revealed that an increase in either moisture content of the medium, sucrose, or NaH_2_PO_4_ resulted in fibrinolytic enzymes production up to optimum level, whereas further increase in concentration decreased the enzyme yield. The perturbation plot (Figure 4) showed that moisture had a significant effect on fibrinolytic enzymes production compared to other variables. The fibrinolytic enzymes production were 4418 units/mL in an optimized medium composed of (%) sucrose (0.5), NaH_2_PO_4_ (0.075), and moisture (113.64). Results revealed that the RSM optimized medium increased 4.5-fold of fibrinolytic enzymes production than the unoptimized medium. Fibrinolytic activities were comparatively higher than the earlier report of RSM on *Bacillus subtilis* [21] and on *Bacillus* sp. strain AS-S20-1 [37].

### 3.6. Validation of the Experimental Designs

To validate the model equation, experiments were carried out in triplicates for fibrinolytic enzymes production in optimized conditions predicted by the experimental model and the fibrinolytic enzymes production was found to be 4683 units/mL. The theoretical predicted response for the model equation was 4720 units/mL. The experimental result was very close to the predicted response which validated this model experimentally.

## 4. Conclusion

A *Paenibacillus *sp. IND8 shows enhanced production of fibrinolytic enzymes in statistically optimized medium. The optimized medium showed high fibrinolytic activities of 4418 units/mL, which is 4.5-fold than that of the unoptimized medium. Purification and characterization of these fibrinolytic enzymes are in progress. This enzyme may be considered as a new source for thrombolytic agents.

## Figures and Tables

**Table 1:** Experimental design and results of the 2^5^ fractorial design.

Run	Sucrose	Yeast extract	NaH_2_PO_4_	pH	Moisture	Response (*Y*)
1	−1	1	−1	1	1	1711
2	−1	−1	1	−1	1	2169
3	1	1	−1	1	−1	1931
4	1	1	1	−1	−1	2297
5	−1	1	1	−1	1	3413
6	−1	1	1	1	−1	1244
7	−1	−1	−1	−1	1	1867
8	1	−1	1	1	1	1995
9	1	1	1	1	−1	1976
10	−1	−1	−1	1	1	2873
11	1	−1	1	−1	−1	723
12	−1	1	−1	−1	−1	787
13	1	−1	−1	−1	1	2681
14	1	−1	−1	−1	−1	2315
15	−1	1	−1	1	−1	1400
16	1	−1	1	1	−1	1400
17	1	1	−1	1	1	3514
18	1	1	−1	−1	1	2086
19	−1	−1	1	1	1	2535
20	1	−1	−1	1	1	2251
21	1	−1	−1	1	−1	2910
22	1	1	−1	−1	−1	1171
23	−1	−1	1	1	−1	1345
24	−1	1	1	1	1	1940
25	1	−1	1	−1	1	3331
26	−1	−1	1	−1	−1	2535
27	−1	1	1	−1	−1	2544
28	−1	−1	−1	1	−1	1381
29	1	1	1	1	1	2123
30	−1	−1	−1	−1	−1	1894
31	−1	1	−1	−1	1	1995
32	1	1	1	−1	1	3768

**Table 2:** ANOVA for selected factorial model.

Source	Sum of squares	df	Mean square	*F* Value	*P* value
Model	1.788*E* + 007	27	6.621*E* + 005	125.57	0.0001
A-sucrose	7.317*E* + 005	1	7.317*E* + 005	138.78	0.0003
C-NaH_2_PO_4_	2.066*E* + 005	1	2.066*E* + 005	39.18	0.0033
D-pH	2.901*E* + 005	1	2.901*E* + 005	55.02	0.0018
E-moisture	4.804*E* + 006	1	4.804*E* + 006	911.14	<0.0001
Residual	21091.13	4	5272.78		
Cor total	1.790*E* + 007	31			

**Table 3:** Variables and their levels for response surface methodology.

Variables	Symbol	Coded levels				
		−1.681	−1	0	+1	+1.681
Sucrose	A	0.079	0.25	0.5	0.75	0.92
NaH_2_PO_4_	B	0	0.01	0.055	0.1	0.13
Moisture	C	46.36	60	80	100	113.64

**Table 4:** Central composite designs matrix and results on the production of fibrinolytic enzymes.

Run	Factor A	Factor B	Factor C	Response (*Y*)
1	1	−1	−1	3398
2	1	1	1	4008
3	0	0	0	3450
4	−1.682	0	0	2903
5	1.682	0	0	2661
6	0	0	0	3360
7	−1	1	1	3335
8	0	−1.682	0	3661
9	0	0	0	3808
10	0	0	1.682	4418
11	1	1	−1	1513
12	0	0	0	3673
13	−1	−1	−1	1520
14	0	0	0	3755
15	1	−1	1	3829
16	0	0	0	4314
17	−1	−1	1	4241
18	−1	1	−1	450
19	0	1.682	0	1713
20	0	0	−1.682	1957

**Table 5:** ANOVA for response surface quadratic model.

Source	Sum of squares	df	Mean square	*F* Value	*P* value
Model	2.109*E* + 007	9	2.344*E* + 006	10.74	0.0005 significant
A-sucrose	5.720*E* + 005	1	5.720*E* + 005	2.62	0.1365
B-NaH_2_PO_4_	3.545*E* + 006	1	5.545*E* + 006	16.25	0.0024
C-moisture	1.176*E* + 007	1	1.176*E* + 007	53.88	<0.0001
AB	9112.50	1	9112.50	0.042	0.8422
AC	8.978*E* + 005	1	8.978*E* + 005	4.11	0.0700
BC	6.205*E* + 005	1	6.205*E* + 005	2.84	0.1226
A^2^	1.706*E* + 006	1	1.706*E* + 006	7.82	0.0189
B^2^	2.055*E* + 006	1	2.055*E* + 006	9.42	0.0119
C^2^	5.805*E* + 005	1	5.805*E* + 005	2.66	0.1339
Residual	2.182*E* + 006	10	2.182*E* + 005		
Lack of fit	1.616*E* + 006	5	3.232*E* + 005	2.85	0.1373 not significant
Pure error	5.662*E* + 005	5	1.132*E* + 005		
Cor total	2.328*E* + 007	19			
